# Spectral density-based clustering algorithms for complex networks

**DOI:** 10.3389/fnins.2023.926321

**Published:** 2023-03-30

**Authors:** Taiane Coelho Ramos, Janaina Mourão-Miranda, André Fujita

**Affiliations:** ^1^Department of Computer Science, Institute of Mathematics and Statistics, University of São Paulo, São Paulo, Brazil; ^2^Department of Computer Science, Centre for Medical Image Computing, University College London, London, United Kingdom; ^3^Max Planck Centre for Computational Psychiatry and Ageing Research, University College London, London, United Kingdom

**Keywords:** clustering, graphs and networks, graph theory, spectral methods, electrocorticography, complex networks

## Abstract

**Introduction:**

Clustering is usually the first exploratory analysis step in empirical data. When the data set comprises graphs, the most common approaches focus on clustering its vertices. In this work, we are interested in grouping networks with similar connectivity structures together instead of grouping vertices of the graph. We could apply this approach to functional brain networks (FBNs) for identifying subgroups of people presenting similar functional connectivity, such as studying a mental disorder. The main problem is that real-world networks present natural fluctuations, which we should consider.

**Methods:**

In this context, spectral density is an exciting feature because graphs generated by different models present distinct spectral densities, thus presenting different connectivity structures. We introduce two clustering methods: k-means for graphs of the same size and gCEM, a model-based approach for graphs of different sizes. We evaluated their performance in toy models. Finally, we applied them to FBNs of monkeys under anesthesia and a dataset of chemical compounds.

**Results:**

We show that our methods work well in both toy models and real-world data. They present good results for clustering graphs presenting different connectivity structures even when they present the same number of edges, vertices, and degree of centrality.

**Discussion:**

We recommend using k-means-based clustering for graphs when graphs present the same number of vertices and the gCEM method when graphs present a different number of vertices.

## 1. Introduction

Functional brain networks (FBNs) (Friston, 2011; Fukushima et al., 2011) are connectivity structures derived from brain recordings such as functional magnetic resonance imaging (fMRI), electroencephalogram (EEG), or electrocorticogram (ECoG) (Sporns et al., 2004; van Straaten and Stam, 2013). The network's vertices represent the brain's regions. The weight of an edge connecting two vertices represents the synchrony of the brain's vertices signals. The most commonly used measure is the Pearson correlation. We infer that regions that activate together may be involved in similar functions. Thus, we assume the FBN captures a functional organization of the brain (Lieǵeois et al., 2020).

**Figure 1:**
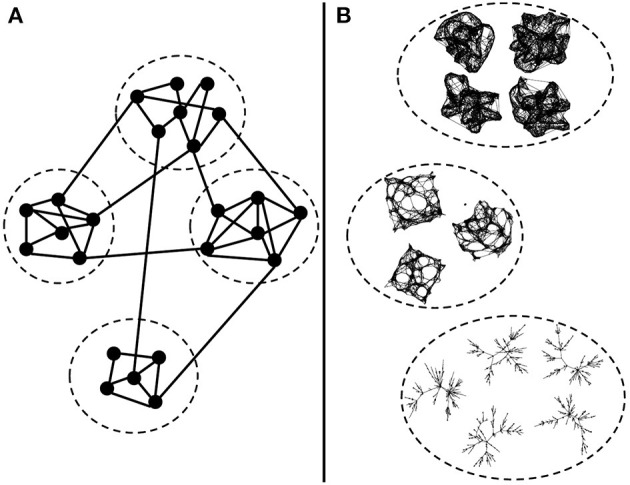
Clustering network strategies. **(A)** Usual graph clustering methods: cluster vertices of a graph into subgraphs. Highly connected vertices form one cluster. **(B)** Our goal: cluster graphs into groups of similar connectivity structures. In this example, we have graphs generated by three different random graph models. Graphs of the same model and set of parameters belong to the same cluster.

It is common to use community detection strategies for identifying sub-networks in the FBNs (Figure 1A). The community detection methods consider the highly interconnected vertices of the same cluster. Examples of algorithms are graph partitioning (Kernighan and Lin, 1970), hierarchical clustering (Friedman, 2017), k-means (MacQueen et al., 1967), and spectral clustering (Spielman and Teng, 2007; von Luxburg, 2007) [for review of methods for clustering a graph's vertices, see Fortunato (2010)].

Another possibility is to group whole FBNs (Figure 1B). The classic approach would be graph kernel methods for measuring the similarity between graphs and a supervised approach for graph classification (Yanardag and Vishwanathan, 2015; Kriege et al., 2019). This method depends on the considered graph centrality measures, which can be a drawback depending on the graph models involved. For example, the degree centrality is the most straightforward measure. However, it does not consider different types of paths involving the vertices. Thus, a method based solely on the degree centrality would not differentiate between a Watts-Strogatz and a k-regular graph because both present vertices with k neighbors.

In this work, we are not interested in clustering vertices of the graph (Figure 1A). We aim to group graphs based on the similarity of their connectivity structure (Figure 1B). We propose graph clustering methods to group whole graphs together based on their graph connectivity structure instead of traditional graph centrality measures.

We know that different random graph models generate different graphs' spectral densities (Bollobás and Béla, 2001; Takahashi et al., 2012). Thus we propose using the graph spectral density (Wilson and Zhu, 2008; Wills and Meyer, 2020) as a resume measure of the graph capable of capturing its connectivity structure. Another advantage of this measure is its independence of vertex correspondence, which helps identify networks presenting similar connectivity patterns (Demirci et al., 2008; Wilson and Zhu, 2008).

Based on the spectral density, we propose a measure of distance and a k-means-inspired algorithm (MacQueen et al., 1967; Lloyd, 1982) to cluster graphs based on the spectral density. However, graphs generated by the same random graph model and parameters, i.e., graphs belonging to the same cluster, may present different sizes. In this case, the spectral densities' supports differ, leading to unreal distances between graphs. Therefore, we will use a model-based approach to cluster graphs of different sizes. The idea is based on the Gaussian mixture clustering approach (Celeux and Govaert, 1992, 1995), where we represent each cluster by a Gaussian distribution. Here we assume that two or more graph models generate the networks. Thus, we assign networks generated by the same model to the same cluster and networks generated by different models to different clusters.

We can use the algorithms proposed here to study many types of complex networks and help understand their connectivity structure. We designed three simulation scenarios with varying graph sizes and random graph models to assess the algorithms' performance. Finally, we illustrate the application of our proposed methods in two contexts: clustering the functional brain networks during anesthesia procedure and clustering two sets of chemical compounds.

## 2. Materials and methods

### 2.1. Goal

Our objective is to cluster graphs that present similar connectivity structures. In other words, we want to group the graphs generated by the same random graph model with the same parameters.

Thus, let *N* and *K* be the number of graphs and clusters. Our goal consists of grouping the *N* graphs into *K* clusters so that graphs generated by the same random graph model belong to the same group.

### 2.2. Graph spectral density

Let *G* = (*V, E*) be a graph composed of a set *V* of |*V*| vertices and a set *E* of |*E*| edges where each edge in *E* connects two vertices in *V*. Any undirected graph *G* can be represented by its |*V*| × |*V*| adjacency matrix **A** where **A**_*ij*_ = **A**_*ji*_ = 1 (*i, j* = 1, …, |*V*|) if vertices *i* and *j* are connected, and 0 otherwise.

The spectrum of *G* is the set of eigenvalues (λ_1_ ≥ λ_2_ ≥ … ≥ λ_|*V*|_) of the adjacency matrix **A**. Notice that since *G* is undirected, **A** is symmetric and consequently (λ_1_, λ_2_, …, λ_|*V*|_) are real. Let λ be the vector composed of all eigenvalues of a given graph *G* and δ be the Dirac delta function. Then the spectral distribution of a random graph *G* is defined as


(1) $$\begin{array}{l} \rho_{G}\left(\text{λ}\right)=\frac{1}{\left|V\right|}\overset{\left|V\right|}{\underset{j=1}{\sum }}\delta \left(\text{λ}-{\text{λ}}_{j}\right). \end{array}$$

To estimate the spectral density (Santos et al., 2021), we use a Gaussian kernel regression with the Nadaraya-Watson estimator (Nadaraya, 1964; Watson, 1964). We set the bandwidth of the Gaussian kernel by (λ_1_ − λ_|*V*|_)/number of bins, and the number of bins by using the Sturges criterion (Sturges, 1926). Finally, we normalize the area below the curve to one.

### 2.3. Distance measures between graph spectra

#### 2.3.1. Kullback-Leibler divergence

As aforementioned, two different spectral densities indicate that different random graph models or sets of parameters generated the graphs. Thus, we can use the spectral density divergence to measure dissimilarity between graphs. Here we use the Kullback-Leibler divergence, hereafter called the KL divergence. Let ρ_*G*_1__ be the spectral density of a given graph and ρ_*G*_2__ be the reference spectral density. Then we define the KL divergence as


(2) $$\begin{array}{l} \begin{array}{l} KL(\rho_{G_{1}}|\rho_{G_{2}})=\;\int_{+\infty }^{-\infty }\rho_{G_{1}}(\text{λ})\log\frac{\rho_{G_{1}}(\text{λ})}{\rho_{G_{2}}(\text{λ})}d\text{λ}, \end{array} \end{array}$$

if the support of ρ_*G*_1__ contains the support of ρ_*G*_2__ and *KL*(ρ_*G*_1__|ρ_*G*_2__) = ∞ otherwise (Takahashi et al., 2012; de Siqueira Santos et al., 2016). To solve this equation computationally, we replace the integral with the Riemann sum. Note that the KL divergence is not a symmetrical measure, i.e., *KL*(ρ_*G*_1__|ρ_*G*_2__) ≠ *KL*(ρ_*G*_2__|ρ_*G*_1__). Thus, the KL divergence is useful in cases where the reference is clear.

#### 2.3.2. Jensen-Shannon divergence

In cases where we wish to compare graphs, but it is unclear which spectral density is the reference, we can use the Jensen-Shannon divergence (JS). Let $\rho_{G_{m}}=\frac{1}{2}\left(\rho_{G_{1}}+\rho_{G_{2}}\right)$. Then we define the JS divergence between two spectral densities ρ_*G*_1__ and ρ_*G*_2__ as


(3) $$\begin{array}{l} JS\left(\rho_{G_{1}},\rho_{G_{2}}\right)=\frac{1}{2}KL\left(\rho_{G_{1}}|\rho_{G_{m}}\right)+\frac{1}{2}KL\left(\rho_{G_{2}}|\rho_{G_{m}}\right). \end{array}$$

The JS divergence is symmetric, i.e., *JS*(ρ_*G*_1__, ρ_*G*_2__) = *JS*(ρ_*G*_2__, ρ_*G*_1__), and non-negative. Also, the square root of the JS divergence satisfies the triangle inequality. Thus, we can use $\sqrt{JS\left(\rho_{G_{1}},\rho_{G_{2}}\right)}$ as a measure of the distance between spectral densities ρ_*G*_1__ and ρ_*G*_2__.

In the following sections, we will describe two clustering algorithms for graphs based on Kullback-Leibler and Jensen-Shannon divergences.

### 2.4. K-means-based graph clustering algorithm

K-means is one of the most popular clustering algorithms due to its simplicity. The algorithm consists of initializing *K* clusters' centroids and iteratively assigning the items to the cluster with the nearest centroid. To adapt k-means for our graphs clustering problem, we will first represent each graph by its spectral density. We define the *K* centroids as the arithmetic mean of all spectral densities belonging to the cluster. The JS divergence gives the distance between the graph and the cluster centroid (see Section 2.3.2). The Algorithm 1 describes the k-means procedure for graphs.

**Algorithm 1:** K-means graph clustering algorithm. **Input**: The *N* graphs and the number of clusters *K*. **Output**: The *K* clusters.

1: *Compute the spectral densities:* For each graph *G*_*i*_, compute its spectral density ρ_*G*_*i*__.
2: *Initialize the clusters:* Let $l_{k}^{G_{i}}=1$ if graph *G*_*i*_ belongs to the *k*th cluster, and $l_{k}^{G_{i}}=0$ otherwise (*k* = 1, …, *K*). Initialize $l_{k}^{G_{i}}$ by randomly assigning each graph *G*_*i*_ to one of the *K* clusters.
3: *Compute the centroid:* $$\begin{array}{l} \rho_{G_{k}}=\frac{\sum_{i=1}^{N}l_{k}^{G_{i}}\rho_{G_{i}}}{\sum_{i=1}^{N}l_{k}^{G_{i}}}. \end{array}$$
4: *Compute the distance of each graph to all clusters:* For each graph *G*_*i*_, compute $$\begin{array}{l} d_{k}^{G_{i}}=\sqrt{JS(\rho_{G_{i}},\rho_{G_{k}}}), \end{array}$$ i.e., the distance between each graph *G*_*i*_ and the *k*th cluster centroid.
5: *Clustering step:* Update $l_{k}^{G_{i}}$ by assigning each graph *G*_*i*_ to the *k*th cluster, which provides the minimum distance ${\text{d}}_{k}^{G_{i}}$.
6: *Recalculate the centroids:* Update ρ_*G*_*k*__ for both clusters that received and lost the graph *G*_*i*_.
7: *Test for convergence:* Go to step 4 until no graph is reassigned.

The k-means is sensitive to the initial condition, i.e., results may change depending on how the algorithm assigns the *N* graphs to the clusters in step 2. Moreover, it uses a greedy search (step 4), i.e., the solution depends on the order of the assignment of graphs to the clusters. Consequently, this method may not find the optimal global solution. Thus, we recommend running the k-means algorithm several times with different initial conditions and selecting the clustering result with the largest silhouette statistic (Rousseeuw, 1987).

### 2.5. Model-based clustering algorithm

Graphs of a different number of vertices have a different amount of eigenvalues. Thus their spectral densities are not directly comparable. For example, suppose we want to classify two graphs generated by an Erdös-Rényi model with *p* = 0.1. In this case, we want to cluster both graphs since the same model generated them. However, if the first graph has 100 vertices and the second has 20 vertices, they would have 100 and 20 eigenvalues, respectively. Thus, their spectral densities would differ due to the number of eigenvalues and not because different models generated them. We know a normalization for some random graph models' spectral density allows comparing graphs of different sizes. For example, we can compare two graphs' spectral densities generated by an Erdös-Rényi random graph model by dividing the eigenvalues by $\sqrt{\left|V\right|}$ (Farkas et al., 2001; Tran et al., 2013). However, the spectral density's normalization is unknown for most random graph models. Thus, we cannot use our k-means-based clustering method for graphs with a different number of vertices. To solve this problem, we propose a model-based method that can cluster correctly even when graphs present a different number of vertices. The idea is inspired by the classification expectation-maximization algorithm. First, we will describe a parameter estimator for random graph models, which will be helpful for the clustering algorithm.

#### 2.5.1. Parameter estimator for graphs

Assuming that a given random graph model generated the graph, we can use the KL divergence to build a parameter estimator. Let *M* be a random graph model, ρ_*G*_ be the spectral density of graph *G*, and {ρ_*M*_(θ)} be a parametric family of spectral densities of the model *M* indexed by a real vector θ of possible parameters for the model. Assume that there is a value θ^*^ of the parameter θ that minimizes the *KL*(ρ_*G*_|ρ_*M*_(θ)). An estimator $\hat{\theta }$ of θ^*^ is given by Takahashi et al. (2012) and de Siqueira Santos et al. (2016)


(4) $$\begin{array}{l} \hat{\theta }=\underset{\theta }{\mathrm{argmin}}KL\left(\rho_{G}|\rho_{M}\left(\theta \right)\right). \end{array}$$

The theoretical spectral density is unknown for most random graph models. Thus, we estimate ρ_*M*_(θ) using a Monte Carlo approach that computes the average spectral distribution of 50 graphs generated by the random graph model *M* with parameter θ.

The parameter estimator based on the KL divergence is implemented in the function graph.param.estimator of the R package statGraph (https://CRAN.R-project.org/package=statGraph).

#### 2.5.2. The expectation maximization-like algorithm

One way to deal with the traditional clustering problem is by applying a model-based method. In this approach, we represent each cluster by a parametric distribution, usually Gaussian. We then model the entire dataset by a mixture of Gaussian distributions and group the items using the expectation-maximization (EM) algorithm (Celeux and Govaert, 1992, 1995). Based on this idea, we propose an EM-inspired method for graphs. We assume that *K* random graph models generate the *N* graphs. Then, we carry out a parameter estimation for each graph using the method described in Section 2.5.1. First, we initialize each cluster's parameters. In the expectation step, we use the KL divergence to measure the dissimilarity between the graph spectral density and each cluster model spectral density. We compute the conditional probability that a graph belongs to a cluster as the inverse of the KL divergence normalized between 0 and 1. We label the graph as belonging to the cluster with the highest conditional probability. In the maximization step, we re-estimate the cluster parameter as an average of the parameters estimated for each cluster's graphs. We repeat the expectation and maximization steps until convergence. This algorithm's outputs are the graph clusters and the estimated model parameters for each cluster. The Algorithm 2 describes the graph classification expectation-maximization (gCEM) approach.

**Algorithm 2:** Graph clustering expectation maximization - gCEM. **Input**: The *N* graphs, the number of clusters *K*, and the *K* random graph models *M*_1_, *M*_2_, …, *M*_*K*_. **Output**: The *K* clusters and the set of estimated parameters for each cluster (random graph model).

1: Initialize the parameters ${\hat{\theta }}_{k}$ of the *K* random graph models *M*_*k*_ (*k* = 1, …, *K*).
2: *Expectation step*: Compute $$\begin{array}{l} t_{k}\left(G_{i}\right)=\frac{1/KL\left(\rho_{G_{i}}|\rho_{M_{k}}\left({\hat{\theta }}_{k}\right)\right)}{\overset{N}{\underset{i=1}{\sum }}1/KL\left(\rho_{G_{i}}|\rho_{M_{k}}\left({\hat{\theta }}_{k}\right)\right)}, \end{array}$$ i.e., the “conditional probability” that graph *G*_*i*_ arises from the *k*th random graph model with parameter ${\hat{\theta }}_{k}$.
3: *Clustering step*: Let *l*_*k*_(*G*_*i*_) be a variable where *l*_*k*_(*G*_*i*_) = 1 if graph *G*_*i*_ belongs to the *k*th cluster, and *l*_*k*_(*G*_*i*_) = 0, otherwise. Update *l*_*k*_(*G*_*i*_) by assigning each graph *G*_*i*_ to the *k*th random graph model, which provides the maximum current *t*_*k*_(*G*_*i*_).
4: *Maximization step*: For each graph *G*_*i*_ in cluster *k*, estimate the parameter ${\hat{\theta }}_{G_{i}}$, assuming that the graph *G*_*i*_ arises from model *M*_*k*_, by using equation 4. Compute $$\begin{array}{l} {\hat{\theta }}_{k}=l_{k}\left(G_{i}\right)\frac{\overset{N}{\underset{i=1}{\sum }}{\hat{\theta }}_{G_{i}}}{\overset{N}{\underset{i=1}{\sum }}l_{k}\left(G_{i}\right)}, \end{array}$$ for *k* = 1, …, *K*.
5: Go to step 2 until convergence of $t_{k}\left(G_{i}\right)\times KL\left(\rho_{G_{i}}|\rho_{M_{k}}\left({\hat{\theta }}_{k}\right)\right)$.

Note that this algorithm may also assume that different random graph models generate each cluster. However, in this case, we propose to run a model selection approach (Takahashi et al., 2012) for each graph.

### 2.6. Random graph models

We use random graph models mainly to study the structural properties of real-world networks. We assume that all graphs are unlabelled, undirected, and without self-loops. In the following sections we briefly describe five random graph models: Erdös-Rényi (Erdös and Rényi, 1959), geometric (Penrose, 1999), k-regular (Bollobás and Béla, 2001), Watts-Strogatz (Watts and Strogatz, 1998), and preferential attachment (Barabási and Albert, 1999).

#### 2.6.1. Erdös-Rényi random graph model

The Erdös-Rényi random graph model (Erdös and Rényi, 1959) consists of generating a graph of |*V*| vertices and connecting each pair of vertices by an edge with probability *p*. An alternative version is uniformly sampling |*E*| edges among all possible edges in the graph.

#### 2.6.2. Geometric random graph model

The geometric random graph model (Penrose, 1999) first generates a graph by uniformly placing |*V*| vertices in the *R*^*d*^ space. Then, it connects two vertices by an edge if their distance is smaller than a radius *r*. In all our experiments, we set *d* = 2.

#### 2.6.3. K-regular random graph model

K-regular random graph model (Bollobás and Béla, 2001) generates graphs where all the |*V*| vertices present the same degree *k*. We call vertex degree the number of edges incident to the vertex. This model generates graphs randomly connecting vertices by trial and error, with the vertices degree being the only constraint.

#### 2.6.4. Watts-Strogatz random graph model

The Watts-Strogatz random graph model (Watts and Strogatz, 1998) generates graphs with |*V*| vertices according to a generative algorithm as follows. First, create a ring lattice with |*V*| vertices connecting each vertex with its *k* nearest neighbors, *k*/2 on each side. Then, for every edge (*i, j*) (where *i* < *j*), with probability *p* replace the edge by (*i, l*), where *l* ≠ *i* ≠ *j* is randomly selected among all vertices. This model creates graphs with “small-world” (high local vertex clustering and small average shortest path) properties, which we observed in many real-world networks. Notice that as parameter *p* → 1, the Watts-Strogatz model approaches an Erdös-Rényi model. Its spectral density also approaches the semicircle distribution typical of the Erdös-Rényi model (Farkas et al., 2001).

#### 2.6.5. Preferential attachment random graph model

Preferential attachment random graph models generate graphs with a power-law (“scale-free”) degree distribution (Barabási and Albert, 1999). New vertices in the network tend to connect to the vertices with the highest degrees. This tendency creates “hub” vertices, i.e., vertices with a very high degree. Preferential attachment models have many implementations, of which the most known is the Barabási-Albert model (Barabási and Albert, 1999). Below we describe the preferential attachment algorithm as implemented in the igraphR package.

Start with *m* ≤ |*V*| vertices in a clique, i.e., all vertices are connected. At each iteration, add a new vertex with *m* connections to the graph's previously added vertices. The probability that the new vertex will choose a given vertex *v*_*i*_ to connect with is proportional to the degree of vertex *v*_*i*_ and the scaling factor *ps* as follows: $P\left(v_{i}\right)\sim \text{degree}{\left(v_{i}\right)}^{ps}$. New vertices are added until the graph contains |*V*| vertices.

### 2.7. Markov random switching algorithm

In simulation 2 scenario (b) described in section 2.8, we wish to gradually disturb a ring lattice's connectivity while preserving network size, density, and degree sequence. To this end, we rewire edges on the original network following the Markov random switching algorithm (de Lange et al., 1995; Maslov and Sneppen, 2002) described as follows. Select a pair of edges (*i*_1_, *j*_1_), (*i*_2_, *j*_2_), where *i*_1_ ≠ *j*_1_ ≠ *i*_2_ ≠ *j*_2_ and try to rewire the edges to become (*i*_1_, *j*_2_), (*i*_2_, *j*_1_). Select a new pair of edges if any new edges already exist in the network.

### 2.8. Simulations

To evaluate the performance of our algorithms, we designed three simulations. In each simulation scenario, we defined the “true cluster” as the set of graphs generated by the same random graph model *M* and the same set of parameters θ. We describe the set-up of the simulations' scenarios as follows:

*1. Graphs of the same size*. This simulation is composed of six scenarios as follows:
  (a) three Erdös-Rényi random graph models with parameters *p*_1_ = 0.2, *p*_2_ = 0.25, and *p*_3_ = 0.3;
  (b) three geometric random graph models with parameters *r*_1_ = 0.15, *r*_2_ = 0.25, and *r*_3_ = 0.35;
  (c) three k-regular random graph models with parameters *k*_1_ = 2, *k*_2_ = 4, and *k*_3_ = 6;
  (d) three Watts-Strogatz random graph models with parameters *p*_1_ = 0.05, *p*_2_ = 0.10, and *p*_3_ = 0.15 and parameter *k* = 16 to all clusters;
  (e) three preferential attachment random graph models with parameters *ps*_1_ = 1, *ps*_2_ = 2, and *ps*_3_ = 3 and parameter *m* = 10 to all clusters;
  (f) and one geometric random graph model with parameter *r* = 0.1, one preferential attachment random graph model with parameters *ps* = 1.5 and *m* = 10, and one k-regular random graph model with parameter *k* = 2.

Notice that there are three clusters in each scenario. We set the number of graphs as 10 per cluster, i.e., a total number of *N* = 30 graphs. To evaluate the graph size's effect on the clustering algorithm, we set |*V*| = 30, 60, 90, 120.

*2. Graphs of the same size and number of edges*. This simulation is composed of two scenarios as follows:
  (a) one Watts-Strogatz random graph model with parameters *k* = 20 and *p* varying from 0 to 1, in steps of 0.1, and one Erdös-Rényi random graph model with parameter |*E*| = 1000. Notice that, as *p* increases, the Watts-Strogatz graphs become more similar to an Erdös-Rényi graph. In the end, the graphs should not be distinguishable.
  (b) start with one Watts-Strogatz random graph model with parameters *p* = 0 and *k* = 20 and one k-regular random graph model with parameter *k* = 20. Then, carry out the Markov random switching algorithm described in section 2.7 to rewire 100, 200, …, 1000 pairs of edges of the Watts-Strogatz graphs. Notice that, as the number of edges rewired increases, the Watts-Strogatz graphs become more similar to a k-regular graph. In the end, the graphs should not be distinguishable.

For both scenarios, there are two initial clusters with different connectivity structures. The first is composed of ring-lattice graphs, and the second may be composed of either Erdös-Rényi or k-regular graphs. We gradually increase perturbations in the connectivity of the ring-lattice graphs while keeping the second cluster parameters fixed. This process makes the Watts-Strogatz graphs approach the structure of the Erdös-Rényi or k-regular graphs. Each cluster is composed of 10 graphs (*N* = 20) with |*V*| = 100 vertices.

3. *Graphs of different sizes*. This simulation comprises six scenarios, as described in simulation 1. The difference is that we sample the size uniformly from the interval [30, 120] for each graph. In other words, graphs belonging to the same cluster may present different sizes.

We ran each simulation's scenario 100 times. We generated graphs independently for each scenario.

### 2.9. Real-world networks

To illustrate the applicability of our clustering algorithms to real-world problems, we describe two datasets.

#### 2.9.1. Functional brain networks

To evaluate the applicability of k-means to real-world data, we used the “anesthesia task” data (Yanagawa et al., 1987) available at the neurotycho project website (http://neurotycho.org/). The goal was to determine when the functional brain network changes its state between awake and under anesthesia. This dataset is composed of 128 (channels) electrocorticogram (ECoG) time series collected from a monkey (*Macaca fuscata*) at a sampling rate of 1 kHz and down-sampled to 200 Hz [see Nagasaka et al. (1911) for further details]. We analyzed the data from experiments performed on two days with the same monkey, Chibi, under the anesthetic agent Medetomidine. To avoid misunderstandings, we call the data from days 1 and 2 Chibi 1 and 2, respectively. First, ECoG data was collected for a few minutes with the monkey awakened and seated with the arms restrained. At the time points 2 581 seconds for Chibi 1 and 2 955 seconds for Chibi 2, an anesthetic injection was applied to the monkey. We defined the time point of loss of consciousness (LOC) (4 292 seconds for Chibi 1 and 4 155 seconds for Chibi 2) as the moment the monkey no longer responded to the touch to its hand or the nostril with a cotton swab. A researcher observed the monkey while under anesthesia and tested if there was a response to physical stimuli. We defined the time point of recovery (7 295 seconds for Chibi 1 and 6 533 seconds for Chibi 2) when the monkey responded to physical stimuli with the same intensity as before the anesthesia. We divided the ECoG time series into a one-second window to construct the monkey's functional brain networks (Rubinov and Sporns, 1910). We calculated the Pearson correlation coefficient among each window's time series, thus obtaining a (128 × 128) correlation matrix per second. We created an adjacency matrix of the graph based on the correlation matrix. We assigned one in the adjacency matrix for correlations greater than a cut-off of 0.5. We zeroed otherwise. We sampled one graph every 10 seconds for our analysis, totaling a time series of 876 and 794 graphs for Chibi 1 and 2, respectively. It is essential to mention that other more powerful measures for studying functional connectivity, such as coherence, phase lag index, and Granger causality, could also be explored. We opted for using correlation to illustrate the application of our clustering algorithms on brain networks.

#### 2.9.2. Chemical compounds

To verify whether gCEM can cluster empirical graphs, we applied it to a dataset to which the clusters are known. We combined two chemical compound datasets, MUTAG and BZR, publicly available at http://graphkernels.cs.tu-dortmund.de. The MUTAG dataset comprises 188 graphs with an average number of vertices of 17.93 ± 4.58 [mean ± standard deviation (sd)], an average number of edges of 39.58 ± 11.39, and an average diameter of 8.21 ± 1.84 edges. The BZR dataset comprises 405 graphs with an average number of vertices of 35.75 ± 7.26, an average number of edges of 76.71 ± 15.40, and an average diameter of 11.65 ± 2.11 edges. The goal is to evaluate whether gCEM can obtain the two clusters composed of graphs from the MUTAG dataset and BZR dataset.

### 2.10. Clustering performance evaluation

The Jaccard index is a commonly used measure for clustering comparison and is present in many packages (Gates and Ahn, 2019). It represents a measure of similarity between the expected and obtained clustering (Levandowsky and Winter, 1971). A Jaccard index close to 1 indicates most elements are in the correct cluster (Tibshirani and Walther, 2005). We computed the Jaccard index using R package clusteval.

## 3. Results

### 3.1. Applications to simulated data

**Figure 2:**
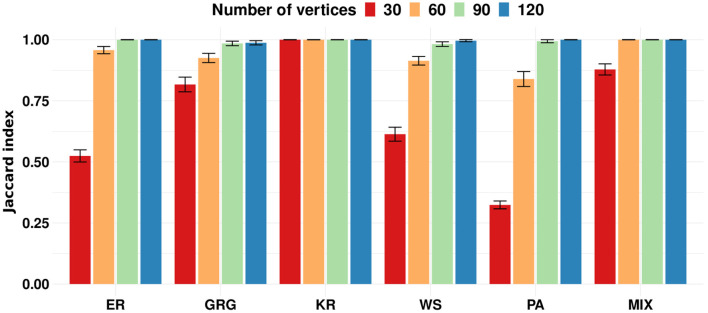
Each group of bars represents a scenario of simulation 1 repeated 100 times for graphs' sizes 30, 60, 90, and 120. The scenarios are composed of the following random graph models: Erdös-Rényi (ER), geometric (GRG), k-regular (KR), Watts-Strogatz (WS), Preferential Attachment (PA), and a mixture of geometric, k-regular, and preferential attachment random graph models (MIX). The y-axis represents the average Jaccard index. Error bars represent 95% confidence intervals. The proposed k-means performs better as the graph size increases.

To evaluate the proposed clustering algorithms' performance, we apply the k-means algorithm to the simulation 1 scenarios described in Section 2.8. In this simulation, each scenario is composed of three clusters. The scenarios from (a) to (e) are composed of three clusters generated by the same random graph model but with different parameters. Scenario (f) is composed of three clusters represented by different models. We designed the simulation scenarios to show that our proposal works correctly on many different types of graph structures. To evaluate if the algorithm performs better as the number of vertices increases, we ran each scenario with |*V*| = 30, 60, 90, and 120. We assume that graphs generated by the same model and parameters belong to the same cluster. Figure 2 shows the results obtained by the k-means on simulation 1. The Jaccard indices increase with the number of vertices in the graphs. In other words, we empirically show that the larger the graph, the better the clustering performance of k-means. It is an expected result because the graph's spectral density converges to an expected density when the number of vertices tends to infinity (McKay, 1981; Farkas et al., 2001; Blackwell et al., 2007; Dumitriu et al., 2012; Tran et al., 2013). Please notice that a dataset of small graphs (|*V*| < 60) might be a limitation for applying the method.

**Figure 3:**
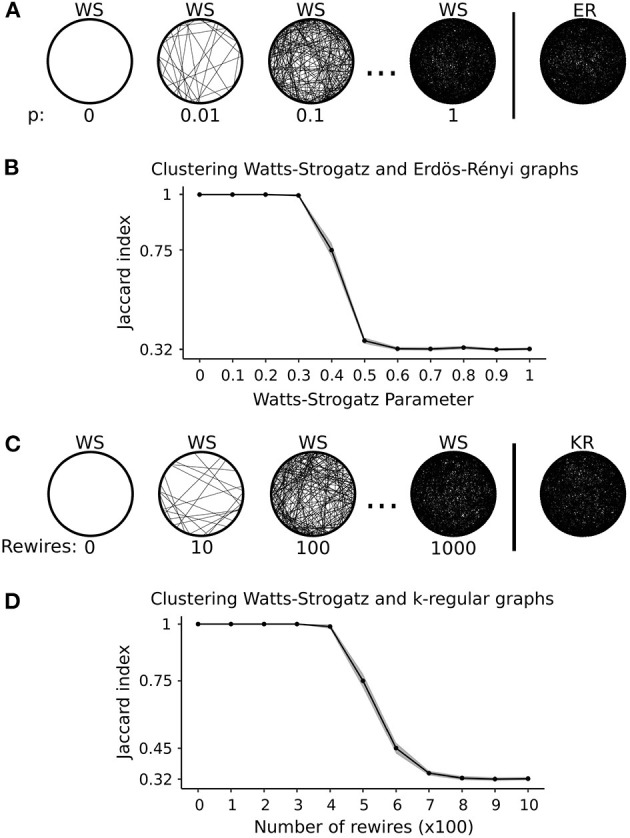
Graphical representation and results of simulation 2. **(A)** Graphical representation of scenario (a), i.e., varying Watts-Strogatz (WS) graphs parameter until it approaches an Erdös-Rényi (ER) graph. **(B)** Results of scenario (a). Each point represents the average Jaccard index of 100 runs. The shaded area represents the 95% confidence interval. **(C)** Graphical representation of scenario (b), i.e., rewiring Watts-Strogatz (WS) graphs edges until it approaches a k-regular (KR) graph. **(D)** Results of scenario (b). Each point represents the average Jaccard index of 100 runs. The shaded area represents the 95% confidence interval. The Jaccard index of 0.32 corresponds to 50% accuracy for both scenarios.

In simulation 2, we are interested in verifying whether the k-means-based method can cluster correctly even when the graphs have the same number of edges. In scenario (a), we start with two clusters: one composed of Erdös-Rényi graphs and the other composed of Watts-Strogatz graphs, all with a fixed number of vertices (|*V*| = 100) and edges (|*E*| = 1000). The Watts-Strogatz parameter started at *p* = 0 (regular ring lattice). In every run, we increased the Watts-Strogatz parameter *p* by 0.1 to increase the edge rewiring until its structure becomes similar to the Erdös-Rényi random graph (*p* = 1). Figure 3A illustrates scenario (a). Figure 3B shows the mean Jaccard index of 100 repetitions for each *p* and the 95% confidence interval. We can see that from *p* = 0 to *p* = 0.3, the k-means correctly clustered all the graphs (Jaccard index = 1). When *p* = 0.4, it misplaces some graphs as represented by the lower Jaccard index. The algorithm classifies the graphs by chance from *p* = 0.5 to *p* = 1. Therefore, this result shows that our proposed k-means for graphs can correctly cluster graphs of the same size and number of edges. However, as the structure becomes similar, it misclassifies as expected.

Besides the number of vertices and edges, another important graph feature is the degree distribution, which may provide enough information to distinguish between clusters. Thus, in simulation 2, we propose scenario (b) that keeps these three features fixed. We start with two clusters: one composed of Watts-Strogatz graphs and the other composed of k-regular random graphs, all with the same number of vertices (|*V*| = 100), edges (|*E*| = 1000), and vertex degree (*d* = 20). In every run, we generate Watts-Strogatz graphs with parameter *p* = 0 and rewire the edges using the Markov random switching algorithm described in Section 2.7. We increase the number of rewires by 100 every run, so the Watts-Strogatz graphs approach k-regular random graphs structure as the number of rewires increases. Figure 3C illustrates the design of simulation 2 scenario (b). Figure 3D shows the mean Jaccard indices of 100 repetitions for each number of rewires on the Watts-Strogatz graphs and the 95% confidence interval. From 0 to 300 rewires, the clustering algorithm could cluster all graphs correctly. At 400 rewires, it misplaced some graphs in some of the 100 repetitions, showing a mean Jaccard index lower than 1. Between 500 and 700 rewires, the algorithm often misplaced some graphs showing lower Jaccard indices. From 800 rewires, the algorithm clustered the graphs by chance.

**Figure 4:**
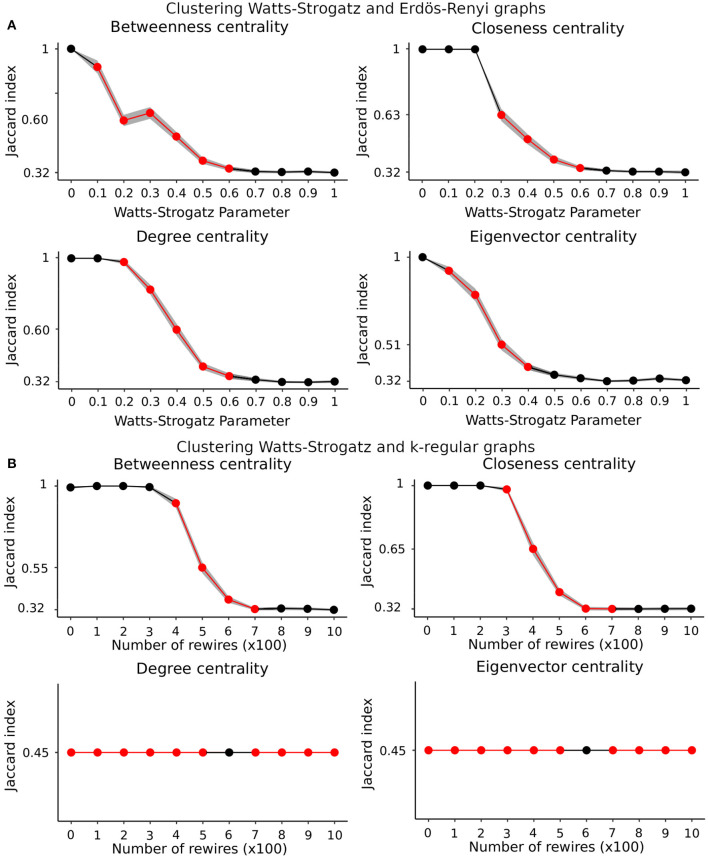
Results of clustering graphs in simulation 2 using betweenness, closeness, degree, and eigenvector centrality measures. **(A)** Results of scenario (a). **(B)** Results of scenario (b). Each point represents the average Jaccard index of 100 runs for both scenarios. The shaded area represents the 95% confidence interval. The Jaccard index of 0.32 corresponds to 50% accuracy. The points in red represent a *p*-value < 0.05 for the ANOVA after Bonferroni correction for multiple tests. In both scenarios, all centrality measures presented a Jaccard index < 1 for lower Watts-Strogatz parameters and fewer rewires compared to results in Figure 3.

Results in Figure 3 show that our proposed k-means-based method for graphs using the spectral distribution as a resume measure could correctly classify graphs with a different connectivity structure. The question of whether we could achieve the same results using graph centralities more commonly adopted in the literature remains. Thus, we ran simulation 2 using four graph centrality measures: betweenness, closeness, degree, and eigenvector centrality. In Figure 4A, with results from scenario (a), we can see that closeness centrality had the best performance, correctly classifying all graphs from *p* = 0 to *p* = 0.2. In panel (B), with results from scenario (b), closeness centrality again had the best performance, correctly classifying all graphs with the number of rewires from 0 to 200. In Figure 4B, neither degree nor eigenvector centrality distinguished the clusters. In other words, the method classified all graphs in the same cluster.

Now we want to test whether the spectral density is a better feature for clustering than centrality measures. We performed ANOVA to compare (Figures 3B, D, 4A, B). We plot the points with a *p*-value < 0.05 after Bonferroni correction for multiple tests in red. The spectral distribution obtained greater classification accuracy in both cases than the centrality-based method. It happened mainly in the interval of approximately [0.2, 0.5] for the Watts-Strogatz parameter and [200, 600] for the number of rewires. The graphs are entirely different for small Watts-Strogatz parameters or fewer rewires. In other words, most clustering methods can correctly group them. On the other hand, for high Watts-Strogatz parameters or a high number of rewires, the graphs become very similar. Thus, most of the clustering algorithms fail.

**Figure 5:**
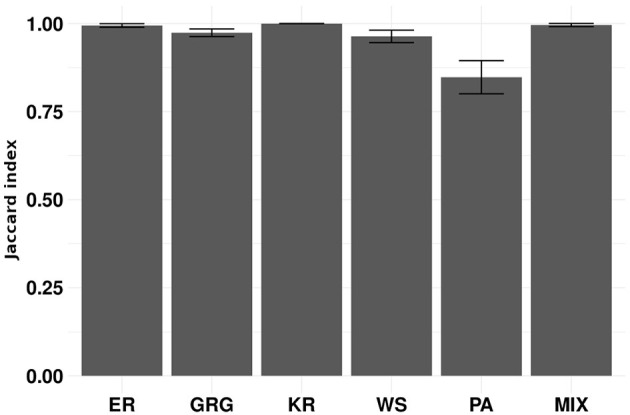
Each bar represents a scenario of simulation 3 in 100 repetitions. We sampled the sizes of the graphs uniformly between 30 and 120. The scenarios are composed of the following random graph models: Erdös-Rényi (ER), geometric (GRG), k-regular (KR), Watts-Strogatz (WS), preferential attachment (PA), and a mixture of geometric, k-regular, and preferential attachment random graph models (MIX). The y-axis represents the average Jaccard indices obtained in 100 repetitions. Error bars represent 95% confidence intervals. The gCEM algorithm clustered graphs correctly, even when the sizes of the graphs are different.

Empirical graphs rarely present the same size. Therefore, we propose the gCEM algorithm, described in Section 2.5.2. We evaluated gCEM using simulation 3 described in Section 2.8. This simulation comprises the same scenarios used in simulation 1. However, we sample the graphs' number of vertices uniformly between 30 and 120. Figure 5 shows that gCEM presents a good performance in all evaluated scenarios. Since the graph's sizes varied from 30 to 120, the expected mean number of vertices is 75. Notice that the results are similar to ours obtained when the graph's sizes are between 60 and 90 in Figure 2.

### 3.2. Applications to empirical data

#### 3.2.1. Functional brain networks

In the anesthesia task dataset, we are interested in identifying when the monkey's functional brain network (FBN) state changes from awake to anesthetized (or vice-versa). In clinical practice, the loss of consciousness (LOC) time point is usually established by responsiveness to auditory or touch stimuli (An et al., 2018). Here we propose to cluster the monkey FBNs into awake or anesthetized groups and identify the monkey's mental state transition points.

Since all the FBNs present the same number of vertices, we applied the proposed k-means. Notice that we have a biological duplicate, i.e., two datasets collected on different days for the same monkey. Thus, we carried out the same analysis on the second day. For each FBN, we obtained a label defining which cluster it belongs to. We adopted label 1 to represent the awakened state and label 0 to represent the anesthetized state. To identify the mental state transition point, we divided the FBNs into two subsets: one referring to the transition from awake to anesthetized (LOC point) and the other referring to the transition from anesthetized to awake (recovery point). We included the FBNs obtained from the experiment's beginning until the anesthetic injection time point for the first subset. We included the same amount of FBNs after the anesthetic injection to keep it as a central reference. For the second subset, we included the FBNs obtained from the time point the researcher certified that the monkey was awake to the experiment's end. We included the same amount of FBNs before the researcher verified that the monkey was awake to keep this point as a central reference. Since the transition between mental states is smooth, we expected to see a time window where the graphs are randomly classified until the transition stabilizes. We estimated the LOC and recovery time points by computing a centered moving average of 50 points on each subset's labels. We predicted LOC and recovery time points as the central point of the window that first resulted in an average lower and higher than 0.5, respectively.

**Figure 6:**
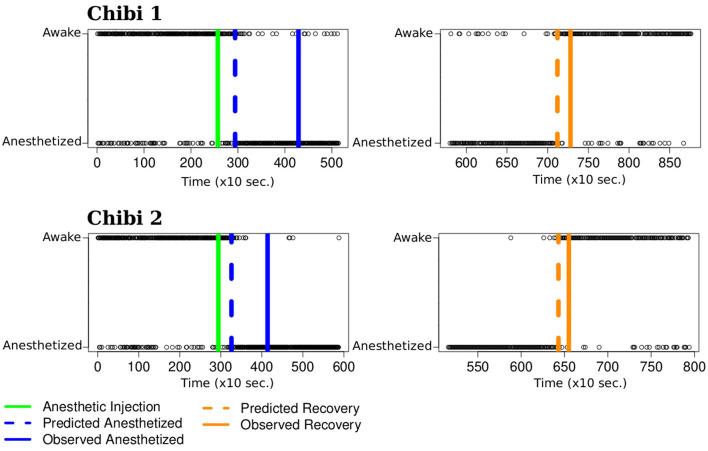
Mental state transition time point. The x and y axes represent the time (in 10 seconds) and the monkey's state. The green line represents the moment of the anesthetic injection. The solid blue line represents when the researcher certifies the monkey stops responding to physical stimuli. The solid orange line represents when the researcher certifies the monkey starts responding again. The black circles are the k-means clustering predictions for the monkey's mental state. We estimate the moment the monkey changes its mental state using a centered moving average. On the **left panels**, it is the time point where the moving average is less than 0.5 (blue dashed line), and on the **right panels**, it is the time point with a moving average greater than 0.5 (orange dashed lines). The predicted changing points occur before the researcher certifies the transition state, suggesting a better prediction from the ECoG signal than the empirical test.

The left panels of Figure 6 represent the first subset of graphs for the transition from awake to anesthetized. The two panels on the right represent the transition from anesthetized to awake graphs. The x and y-axes represent the time and the clustering label, respectively. Each time point contains a corresponding FBN for the monkey represented by a black circle indicating its predicted mental state at that moment. The solid green vertical lines on the left panels represent the moment of the anesthetic injection. The blue solid vertical lines represent when the researcher certified the monkey stopped responding to physical stimuli. The dashed blue vertical lines represent our predicted LOC time point. We observe that the estimated LOC time point is after the anesthetic injection point and before the researcher certifies the monkey is anesthetized. The predicted LOC time point precedes the moment the researcher indicated that the monkey did not respond to external stimuli by 22 minutes for Chibi 1 and 14 minutes for Chibi 2. On the right panels, the solid orange vertical lines represent the moment the researcher certified the monkey started responding to physical stimuli again with the same intensity before the anesthetic injection. The orange dashed lines represent our predicted time point of recovery from the anesthesia. The predicted time point precedes the moment the researcher indicates the monkey was awake by 3.8 minutes for Chibi 1 and 2.5 minutes for Chibi 2. Our results align with expectations and estimate when the monkey's brain state's change occurs before the researcher's observations.

To verify whether our results are robust to the adopted cut-off in constructing the adjacency matrix (Garrison et al., 2011) (the cut-off was 0.5, as described in section 2.9), we experimented with a cut-off of 0.45 and 0.55. Our results and conclusions did not change (see Supplementary Figure S1).

#### 3.2.2. Chemical compounds

**Figure 7:**
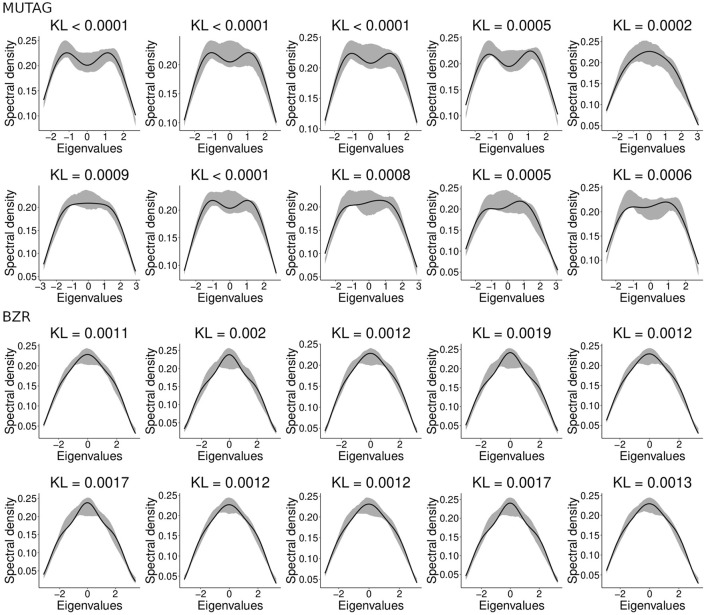
The adjusted Watts-Strogatz spectral densities for 20 randomly selected chemical compounds. The black line represents the compound's spectral density. The shaded area represents the 95% confidence interval for the average spectral densities generated by the Watts-Strogatz model and estimated parameters. The indicated KL represents the divergence between the adjusted Watts-Strogatz model and the chemical compound. Notice that most of the chemical compounds' spectral densities (black line) belong to the gray area, indicating that the Watts-Strogatz model has a good fit for these graphs.

Here we combined two different chemical compound datasets, namely MUTAG and BZR. For further details about these datasets, see Section 2.9. We aim to evaluate whether the clustering algorithm can identify from which dataset each chemical compound came. Since the compounds' graphs present different vertices, we used gCEM. To define which random graph model best fits the chemical compounds, we applied the model selection approach described in Takahashi et al. (2012). The model selection approach consists of choosing the model that minimizes the KL divergence between the spectral densities of the graph and the model. The model selection approach chose the Watts-Strogatz random graph model for 96.12% of the chemical compounds (the model selection approach classified the remaining 23 compounds as Erdös-Rényi). This result does not mean that the Watts-Strogatz model is the one that generated the molecular networks, but instead that it is the best one among the options evaluated. We analyzed the spectral densities obtained by our adjusted network and the actual chemical compound to verify whether the Watts-Strogatz model is a good representation of the chemical compounds. Figure 7 presents an illustrative sample of 20 chemical compounds and the adjusted spectral densities. The gray zone is the 95% confidence interval for the adjusted spectral density. Notice that the Watts-Strogatz model presents a good fit for actual data.

Since we know the true labels, i.e., from which dataset came from each graph, we can measure the clustering performance. The gCEM algorithm correctly classified 536 out of 593 molecules (90.38% accuracy, Jaccard index = 0.7255). The estimated parameters were *p*_1_ = 0.122 for the MUTAG dataset and *p*_2_ = 0.368 for the BZR dataset. The Watts-Strogatz random graph model parameter is not associated with the number of vertices nor the graph's number of edges. Rather, it indicates the randomness of the edges' disposal. The smaller the parameter, the more likely the graph will exhibit small-world properties. Based on the obtained results, we can infer that the MUTAG molecules present more small-world properties than BZR. In contrast, the BZR molecules present a more random configuration than MUTAG.

## 4. Discussion

We propose a method capable of clustering graphs based on their connectivity structure. For this, we first discussed spectral density as a resume measure of the graph. Then, we showed two metrics of the distance between spectral densities: the Kullback-Leibler (KL) and the Jensen-Shannon (JS) distance. Having a distance measure between the graphs, we adapted the traditional k-means clustering algorithm for using the JS as a distance measure and the mean spectrum of all the graphs in each cluster as the cluster centroid.

The spectral distribution shape changes according to the number of eigenvalues. A graph will have the same number of eigenvalues as the number of its vertices. Because of this, we can not generate a mean spectral distribution with graphs of a different number of vertices. Thus, our proposed k-means for graphs have the limitation that all graphs must present the same number of vertices. Then, we proposed the model-based gCEM algorithm as an adaptation of the expectation-maximization clustering algorithm. We used the KL as the distance measure. Also, we generated the model spectrum as the mean spectrum of 50 graphs of the model with the same size as the one we wish to compare with the model.

We designed some simulation scenarios to verify our proposed algorithms' performance. In simulation 1, we showed that the k-means for graphs could correctly cluster graphs with different connectivity. In simulation 2, we showed that the algorithm could correctly cluster graphs even when they present the same number of vertices, edges, and degree distribution. This second simulation scenario is essential to confirm that the spectral distributions capture information about the connectivity structure of the graph and is more powerful than more commonly used graph centrality measures (e.g., betweenness, closeness, degree, and eigenvector centralities). In simulation 3, we show that gCEM can separate graphs with different numbers of vertices.

Finally, we applied our k-means for graphs algorithm to an anesthesia task dataset. We showed that our algorithm separated most awake brain networks from the anesthetized brain networks. To cluster networks of different sizes, we used gCEM to discriminate between two sets of chemical compounds. The networks presented different sizes within and between the groups. As expected, gCEM correctly separated most networks into their original sets.

Our results show that spectral density is a sound graph resume measure for capturing the connectivity structure of graphs. The KL and JS distance measures effectively compare graphs, and our clustering methods work well on multiple graph models. In this way, we recommend considering spectral density as a graph measure in studies aiming to characterize graphs based on connectivity structure. Regarding applying our clustering methods to study FBNs, we recommend using k-means-based clustering for graphs presenting the same number of vertices and gCEM clustering when the networks present a different number of vertices.

Comparing our methods with the classic kernel methods for graph classification (Yanardag and Vishwanathan, 2015; Kriege et al., 2019), our methods have the advantage of not defining the kernel to be used. Both our methods always rely on the spectral density that showed to capture information about the graph structure. Comparing our two methods against each other, the advantages of k-means include being faster and independent of graph model estimation. The disadvantage is not being able to cluster graphs of different sizes. On the other hand, the gCEM advantage is clustering graphs of different sizes and providing estimations for each cluster's parameters, which aids in interpreting the graphs' structural changes among clusters. One limitation is defining one or more random graph models that fit the data.

Although both algorithms tend to converge in a few iterations, the computational time for computing the spectral density distribution for each graph is *O*(|*V*|^3^). Moreover, the parameter estimator (see Section 2.5.1) used in gCEM relies on a Monte Carlo approach. Finally, the gCEM initialization step can be very time-consuming due to computing the spectral density of each graph. Considering all these bottlenecks, applying gCEM is limited to graphs composed of thousands of vertices. One way to run gCEM in more extensive graphs would be to parallelize the spectral density and parameter estimation processes. Also, it is crucial to notice that we are not considering the eigenvectors of the graph in our analysis. However, they also contain essential information. Investigating the relationship between eigenvectors and graph structure in the future might be interesting.

The clustering codes are available at the R package statGraph (https://CRAN.R-project.org/package=statGraph), functions kmeans.graph and gCEM, under the GNU GPL.

## Data availability statement

The datasets analyzed for this study can be found on the Neurotycho project website (http://neurotycho.org/) and at the TUDatasets repository (http://graphkernels.cs.tu-dortmund.de).

## Ethics statement

All the animal experiments and surgical procedures were performed in accordance with the experimental protocols [No. H24-2-203(4)] approved by the RIKEN ethics committee and the recommendations of the Weather all report. The use of non-human primates in research.

## Author contributions

TR and AF conceived the analyzes. TR conducted the analyzes and wrote the first draft of the manuscript. TR, JM-M, and AF analyzed the results and revised the manuscript. All authors read and approved the final version of the manuscript.
