# Supplementary material for: Spectral density-based clustering algorithms for complex networks
Source: Front Neurosci. 2023 Mar 30;17:926321. doi: 10.3389/fnins.2023.926321 (PMC10101435; doi:10.3389/fnins.2023.926321)
Supplement: Supplementary file 1 [file Image_1.pdf]

frontiers

Supplementary Material

1 SUPPLEMENTARY FIGURE

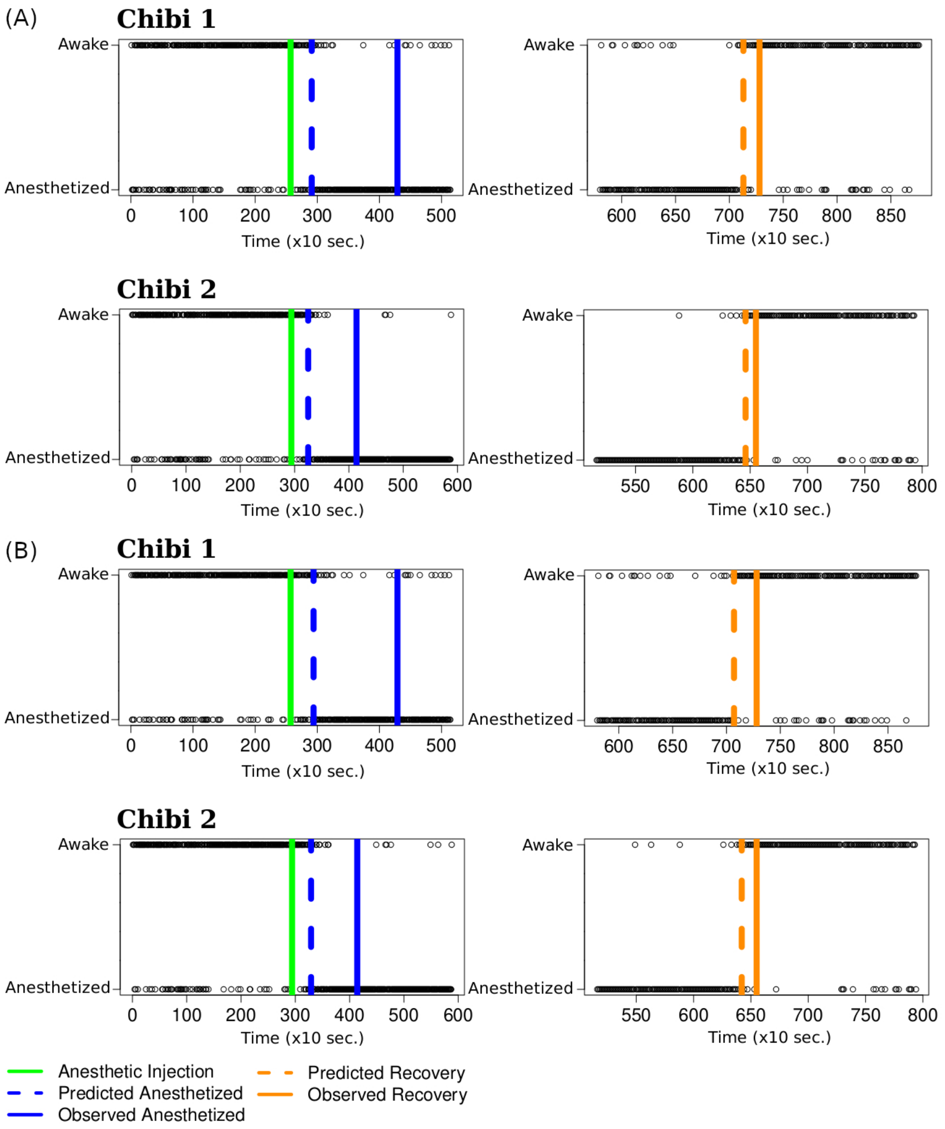

Figure S1. Chibi's mental state transition time point using a cut-off equals 0.45 (panel A) and 0.55 (panel B) to include an edge between two vertices in the graphs. The x and y axes represent the time (in 10 seconds) and the monkey's state. The green line represents the anesthetic injection moment. The solid blue line represents when the researcher certifies that the monkey stops responding to physical stimuli. The solid orange line represents the moment the researcher certifies that the monkey starts responding again. The black circles represent the predicted state of the monkey by k-means clustering. We estimate the moment the monkey changes its mental state as the time point where the centered moving average is less than 0.5 for the left panels (blue dashed line) and greater than 0.5 for the right panels (orange dashed lines). The predicted changing time points are before the researcher certifies the transition state. This result suggests a better prediction from the ECoG signal than the empirical test. Thus, our analytical procedure is robust to slight change (eps = 0.05) in the adopted cut-off for generating the graphs.
